# Plasma Extracellular Vesicles in Children with OSA Disrupt Blood–Brain Barrier Integrity and Endothelial Cell Wound Healing In Vitro

**DOI:** 10.3390/ijms20246233

**Published:** 2019-12-10

**Authors:** Abdelnaby Khalyfa, David Gozal, Leila Kheirandish-Gozal

**Affiliations:** Child Health Research Institute, Department of Child Health, University of Missouri School of Medicine, Columbia, MO 65201, USA; khalyfaa@missouri.edu (A.K.); gozald@health.missouri.edu (D.G.)

**Keywords:** pediatric OSA, extracellular vesicles (EVs), exosomes, neurocognitive deficits, wound healing, hCMEC/D3 cells, electric cell–substrate impedance sensing (ECIS)

## Abstract

Pediatric obstructive sleep apnea (P-OSA) is associated with neurocognitive deficits and endothelial dysfunction, suggesting the possibility that disruption of the blood–brain barrier (BBB) may underlie these morbidities. Extracellular vesicles (EVs), which include exosomes, are small particles involved in cell–cell communications via different mechanisms and could play a role in OSA-associated end-organ injury. To examine the roles of EVs in BBB dysfunction, we recruited three groups of children: (a) absence of OSA or cognitive deficits (CL, *n* = 6), (b) OSA but no evidence of cognitive deficits (OSA-NC(−), *n* = 12), and (c) OSA with evidence of neurocognitive deficits (OSA-NC(+), *n* = 12). All children were age-, gender-, ethnicity-, and BMI-z-score-matched, and those with OSA were also apnea–hypopnea index (AHI)-matched. Plasma EVs were characterized, quantified, and applied on multiple endothelial cell types (HCAEC, HIAEC, human HMVEC-D, HMVEC-C, HMVEC-L, and hCMEC/D3) while measuring monolayer barrier integrity and wound-healing responses. EVs from OSA children induced significant declines in hCMEC/D3 transendothelial impedance compared to CL (*p* < 0.001), and such changes were greater in NC(+) compared to NC(−) (*p* < 0.01). The effects of EVs from each group on wound healing for HCAEC, HIAEC, HMVED-d, and hCMEC/D3 cells were similar, but exhibited significant differences across the three groups, with evidence of disrupted wound healing in P-OSA. However, wound healing in HMVEC-C was only affected by NC(+) (*p* < 0.01 vs. NC(−) or controls (CO). Furthermore, no significant differences emerged in HMVEC-L cell wound healing across all three groups. We conclude that circulating plasma EVs in P-OSA disrupt the integrity of the BBB and exert adverse effects on endothelial wound healing, particularly among OSA-NC(+) children, while also exhibiting endothelial cell type selectivity. Thus, circulating EVs cargo may play important roles in the emergence of end-organ morbidity in pediatric OSA.

## 1. Introduction

Sleep is a fundamental physiological function involved in the restoration of cellular and organ homeostasis and is essential for optimal daytime functioning. Restricted or poor-quality sleep has been associated with neurobehavioral and cognitive deficits, end-organ dysfunction, and chronic health conditions [1,2,3,4,5,6]. Pediatric obstructive sleep apnea (P-OSA) may affect up to 3%–4% of all school-aged children, and is characterized by recurrent episodes of upper airway collapsibility during sleep that leads to disruption of alveolar ventilation, episodic hypoxemia, and sleep fragmentation. A large body of evidence has reported the presence of associations between childhood OSA and behavioral and cognitive problems, including impulsivity, hyperactivity, and decreased attention and academic performance [7,8,9,10,11,12,13,14]. The biological plausibility of these associations has been corroborated by extensive studies in rodent models [15,16,17].

The blood–brain barrier (BBB), a multicellular vascular entity that operates as an active or passive diffusion barrier, regulates the transfer of most compounds in the circulation to the central nervous system [18,19]. Highly differentiated endothelial cells line the walls of the brain capillaries and serve as the primary constitutive elements of the BBB, with the combined surface area of these capillaries forming the major interface for blood–brain exchange [18,20]. Impairments of the endothelial barrier function have been linked to a variety of disease states, and OSA is clearly one of such conditions in which the endothelium emerges as a target organ [21,22,23]. However, the exact mechanisms by which OSA causes neural deficits remain unexplored [24,25]. Identification of biomarkers and of specific endothelia cell selectivity in children with neurocognitive impairments would potentially provide mechanistic insights and therapeutic targets.

Recently, we and others have proposed that perturbations in the BBB may foster the occurrence of cognitive impairments in pediatric OSA [26,27]. Several lines of evidence have uncovered that specific diseases or stressful conditions can induce disruption of BBB endothelial tight junctions and affect cognitive ability. In fact, it is suggested that such processes can be mediated, at least in part, by exosome-related biological activities directly altering the BBB. At a cellular level, we and others found that OSA likely causes cognitive impairments through intermittent hypoxia and sleep fragmentation that promote oxidative stress and systemic inflammation, either independently or via the presence of endothelial dysfunction (ED) [28,29,30]. Exosomes, a class of extracellular vesicles, are very small vesicles ranging in size from 30 to 120 nm. These vesicles are generated by many, if not all, cells in the body, and can perhaps be detected in all biological fluids [27,31,32,33,34,35], where they participate in a vast array of physiological processes, and more specifically, mediate critical aspects of intercellular communication [36] through delivery of their functional cargo [31,34,37,38,39,40].

Studies have shown that perturbations in BBB structures, along with accompanying neurovascular unit dysfunction, have mechanistic roles in the pathogenesis of neurodegenerative diseases, such as Alzheimer’s disease [41]. In parallel with such findings, improved understanding of the mechanisms underlying OSA-induced endothelial and cognitive dysfunction is critical. Here, we expanded on our initial observations on exosomes in pediatric OSA in the context of the BBB [27], and postulated that plasma exosomes derived from children with OSA would differentially affect endothelial cells originating from various vascular beds and their repair capacity. To this effect, we examined brain hCMEC/D3 cells, which constitute the endothelial cells of the BBB, as well as several other endothelial cells such as skin HMVEC-d, coronary artery HCAEC, iliac artery HIAEC, cardiac ventricles HMVEC-C, and lung HMVEC-L.

## 2. Results

### 2.1. Subject Characteristics

**Figure 1:**
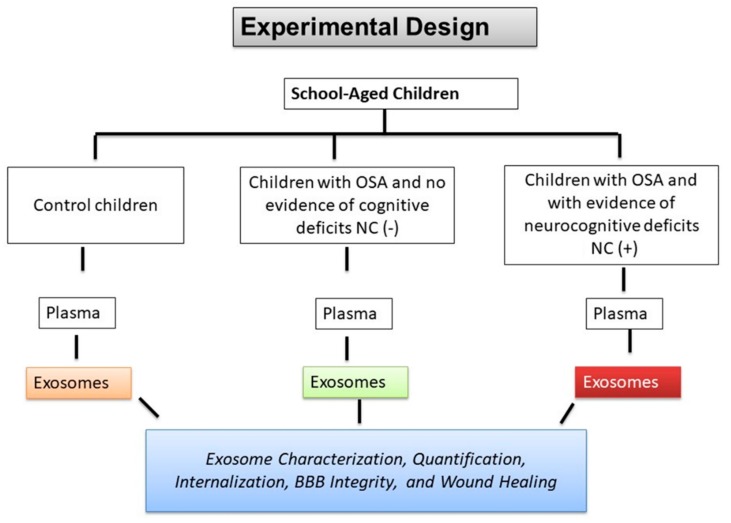
Schematic Illustration of experimental design for school-aged children. A total of 30 children matched for gender, age, BMI z-score, and ethnicity were divided into three groups: control children with normal sleep studies and no evidence of cognitive deficits in neuropsychological standardized tests (*n* = 6), children with OSA and no evidence of cognitive deficits (OSA-NC(−); *n* = 12), and children with OSA and with evidence of neurocognitive deficits (OSA-NC(+); *n* = 12).

In this study, we enrolled a total of 30 children with OSA (*n* = 24) including age-, sex-, ethnicity-, and BMI-z-score-matched controls (*n* = 6). All 30 children underwent nocturnal polysomnography (NPSG) and neurocognitive testing. The demographic and polysomnography characteristics for those 24 subjects are shown in Figure 1 and Table 1, indicating that no significant differences existed except for the presence (NC(+)) or absence (NC(−)) of cognitive dysfunction.

### 2.2. EVs Characterization and Quantification

**Figure 2:**
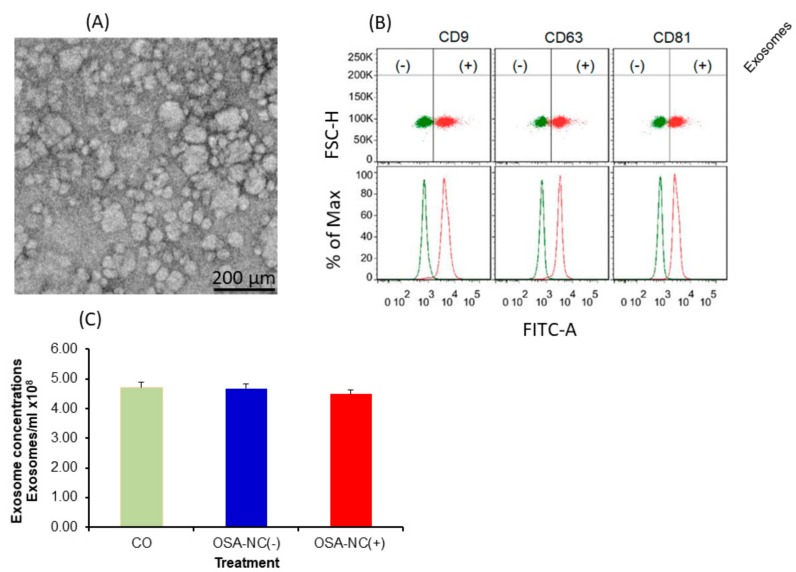
EVs characterization and quantification. Plasma EVs size distribution was determined using electron microscopy. EVs were also characterized using flow cytometry, and quantified using a commercial kit. Panel (**A**) shows the size distribution of EVs. Panel (**B**) shows flow cytometry analysis of purified plasma EVs following specific isolation with magnetic beads stained with anti-CD9, CD63, and CD81. Panel (**C**) The Exo-Flow magnetic stand for EVs separation and FACS analysis shows the presence of EVs (positive, blue color) and absence of EVs (negative, red color); beads are displayed on the FACS plot (*n* = 6).

Plasma EVs isolation and characterization from blood samples were performed using a previously described protocol that enriches for exosomes [6,42]. Indeed, negative stain electron microscopy showed vesicles in typical shaped morphology (Figure 2A) [32,38,43], and as shown in Figure 2B, unique exosome markers were used to conclusively identify EVs using flow cytometry of isolated EVs derived from CO, OSA-NC(−), or OSA-NC(+). As shown in Figure 2B, each image was stained separately with a different antibody using two different negative controls (all reagents without antibody and no EVs (negative #1), all reagents without EVs (negative #2)). Next, we subtracted the mean fluorescent intensity (MFI) of negative #1 from each MFI for each antibody, and the resultant MFIs were as follows: CD9 (356 ± 44.5), CD81 (324 ± 32.2), and CD63 (320 ± 29.21) without exosomes, while MFIs for the samples with exosomes were: CD9 (3794 ± 354), CD81 (2789 ± 241), and CD63 (2201 ± 199), respectively (*n* = 6).

Quantification of EVs showed no significant differences in the number of EVs derived from CO (4.14 ± 0.32 × 10^8^/mL), OSA-NC(−) (4.25 ± 0.35 × 10^8^/mL), or OSA-NC(+) (4.31 ± 0.41 × 10^8^/mL) (Figure 2C).

### 2.3. EVs Uptake

**Figure 3:**
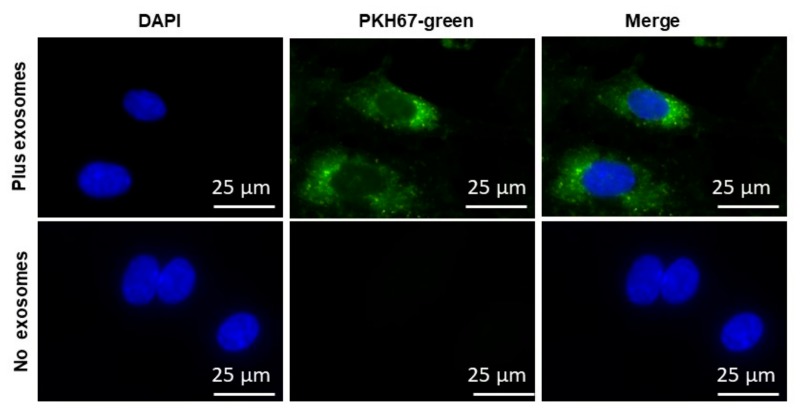
Illustrative example of EVs uptake by naïve human brain microvascular endothelial cells (hCMEC/D3) (*n* = 6 experiments). Confocal microscope images illustrating EVs uptake into hCMEC/D3. EVs were isolated from plasma of children with OSA-NC(+) and labeled with the PKH67 Green Fluorescent (lipophilic). A representative image of hCMEC/D3 cells that were grown on coverslips for 24 h, and the labeled EVs with PKH67 were added to the cells for 24 h at 37 °C. Cells were washed and stained with nuclei (blue) stained with DAPI, *n* = 6, scale bar in 25 µm. As controls, no EVs were used but PKH67 was added. The differential interference contrast (DIC) was used to visualize the morphology of the cells without fluorescence.

Next, we used EVs isolated from human plasma to test whether the EVs that were isolated were internalized into human naïve endothelial cells from various vascular beds. The cell culture media was supplemented individually with PKH67-labeled EVs, *n* = 6 (Figure 3). As shown in the Figure 3, the PKH67 signal was detected in the membrane of cells grown in medium supplemented with PKH67-labeled exosomes, whereas no signal could be detected in cells grown in medium without EVs but in which PKH67 was also added. The imaging approaches corroborated that circulating EVs derived from all children contained active vesicles. No differences emerged in EVs uptake between the various cell types [44]. EVs internalization into endothelial cells was also dose-dependent, with increased spot numbers, total fluorescence, and median pixel intensities of the dyes paralleling increasing EVs concentrations [44]. In this experiment, the same type and volume of EVs were used but different concentrations were employed.

### 2.4. Effects of Circulating EVs on Endothelial Monolayer Barrier Integrity

**Figure 4:**
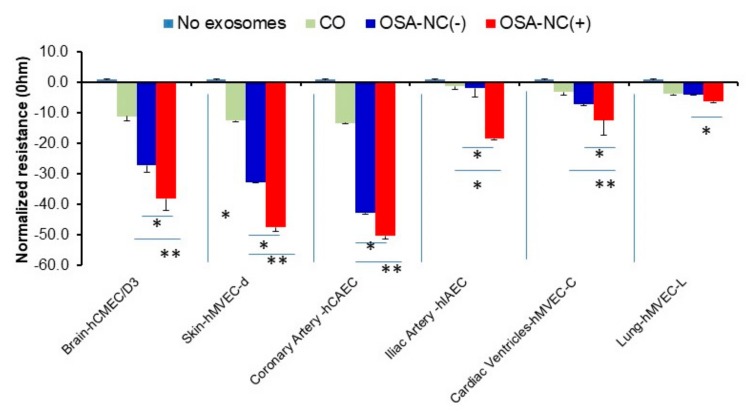
Effects of plasma EVs derived from healthy (CO), OSA-NC(−), and OSA-NC(+) children on endothelial monolayer barrier integrity using endothelial cells originating from multiple vascular beds. Individual cells were seeded at 0 h at density of 50,000 cells into ECIS system arrays (8W10E) for 24 h, then EVs derived from CO, (*n* = 6), OSA-NC(−), (*n* = 12), and OSA-NC(+), (*n* = 12) were added and monitored for another 48 h. * indicates statistical significance, * *p* < 0.01, ** *p* < 0.001. Data was recorded by an ECIS Z-theta instrument equipped with ECIS software.

The effects of plasma EVs derived from healthy (CO), OSA-NC(−), and OSA-NC(+) children on endothelial monolayer barrier integrity revealed differences across conditions which also exhibited vascular bed endothelial cell selectivity. In general, EVs derived from children with OSA-NC(+) showed higher disruption of hCMEC/D3 endothelial cell monolayer barrier integrity compared to children with OSA-NC(−) (*p* = 0.01), as well as compared to CO (*p* < 0.01; Figure 4). Similar results were obtained in hMVEC-d cells and in coronary artery hCAEC cells. However, the effects of EVs on iliac artery hIAEC, cardiac ventricles hMVEC-C, and lung hMVEC-L were markedly attenuated compared to the aforementioned endothelial cells, even if significant differences persisted between EVs derived from OSA-NC(+) compared to OSA-NC(−) or CO among iliac artery hIAEC and cardiac ventricles hMVEC-C cells (Figure 4).

### 2.5. Effects of Circulating EVs on Wound Healing

**Figure 5:**
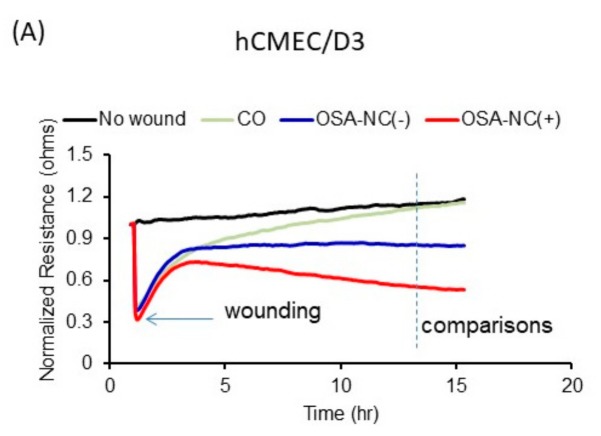

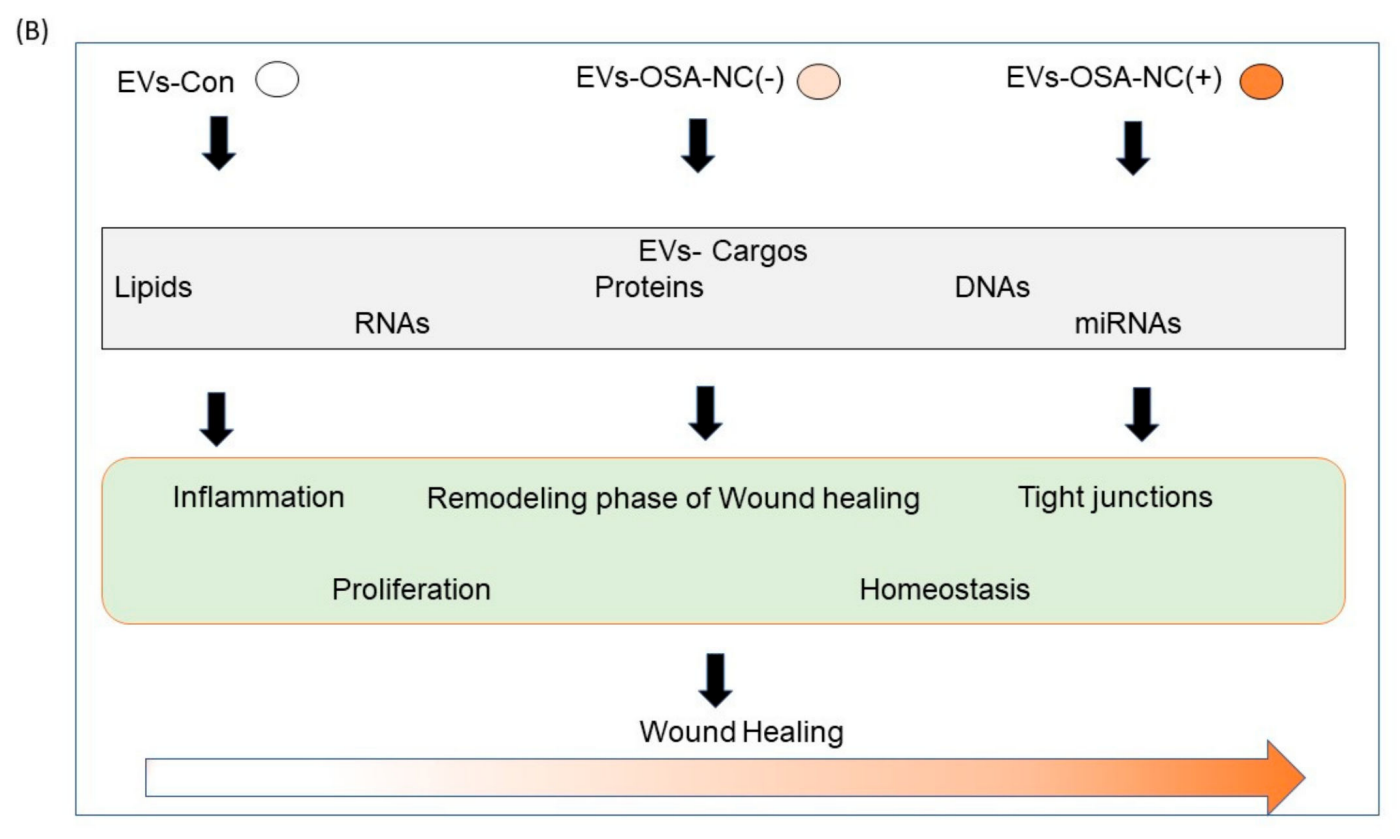
Effects of plasma EVs derived from healthy (CO), OSA-NC(−), and OSA-NC(+) children on endothelial cell migration and recovery following wounding using hCMEC/D3 cells. Panel (**A**) shows representative graphs of comparative analysis of hCMEC/D3 cells treated with and without EVs following wound healing. hCMEC/D3 cells were seeded at 0 h at a density of 50,000 cells into ECIS system arrays (8W10E) for 24 h, cells were wounded, then EVs derived from CO (*n* = 6), OSA-NC(−) (*n* = 12), and OSA-NC(+) (*n* = 12) were added and monitored for another 48 h. Panel (**B**) is a schema that illustrates the effects of EVs cargos derived from healthy (CO), OSA-NC(−), and OSA-NC(+) children on endothelial cell wound healing and recovery of hCMEC/D3 cells.

Figure 5 shows the effects of wound healing on hCMEC/D3 cells treated with EVs derived from healthy (CO), OSA-NC(−), and OSA-NC(+) children (Panel A). We also show in Figure 5 (Panel B) a schema that illustrates the effects of EVs cargos on the recovery of hCMEC/D3. As shown in Table 2, the effects of plasma EVs on wound-healing recovery using endothelial cells originating from multiple vascular beds revealed differences across the three subject groups, as well as differences among the various cell types. Indeed, EVs derived from OSA-NC(+) applied to hCMEC/D3 exhibited much slower recovery (45.08% ± 3.59%) when compared to OSA-NC(−), (71.87% ± 5.37%; *p* = 0.001), while for hMVEC-d, recovery was 79.92% ± 4.25% for OSA-NC(+) compared to 82.97% ± 5.64% in OSA-NC(−) subjects (*p* = 0.01). No statistically significant changes between the three subject groups emerged among the other endothelial cell types (Table 2). As indicated in Table 2, shortly after the electrical pulse used to induce the standard wound, living cells located around the sensing electrode migrated inward to cover the wounded area until the wound was sealed, a process that requires several hours.

## 3. Discussion

In this study, we showed that circulating plasma EVs derived from children with OSA in the presence or absence of cognitive deficits disrupt the integrity of the BBB with such effects being particularly prominent among the children with cognitive deficits. We further showed that the effects of plasma-derived EVs from each of the groups differ across endothelial cells originating from various vascular beds, indicating differential susceptibility of the endothelium as an end-organ target of OSA. Furthermore, endothelial wound healing is cell- and subject group-dependent, with more prominent delays in recovery among children with OSA who display evidence of detectable cognitive deficits. Thus, circulating EVs cargo may play important roles in the emergence of end-organ morbidity in pediatric OSA that is vascular bed selective.

Untreated OSA children are at high risk of neurocognitive deficits particularly affecting specific domains [45,46], and such susceptibility manifests disease severity-dependent features [12]. Furthermore, OSA independently promotes the risk of other functional perturbations such as those affecting lipid and insulin homeostasis and systemic blood pressure regulation [47,48,49,50,51]. In a previous study, we showed that cognitive and vascular deficits were highly likely to overlap, suggesting that the endothelium may serve as the primary target for OSA-associated morbidities [52].

The endothelial BBB cells have highly stringently sealed cell-to-cell contacts that result in high transendothelial electrical resistance along low paracellular and transcellular permeability [53]. Maintaining the BBB integrity is critical for homeostatic regulation and functioning of the chemical characteristics of brain interstitial fluid [54], and plays a critical role in intercellular communication and homeostasis of the central nervous system (CNS) microenvironment by exquisite control of virtually every molecule across the membrane between blood and CNS [55]. Thus, dysfunction of the BBB is likely involved in the pathogenesis of multiple neurological diseases, including the neurocognitive profile of children with OSA [27,52,56]. However, BBB endothelial cells differ from all other endothelial cells by the absence of any fenestrations, the presence of more extensive tight junctions (TJs), and evidence of sparse pinocytic vesicular transport [57]. Tight junction protein complexes underlie critical roles in neuronal homeostasis by restricting the diffusion of ions and molecules across the BBB, and by enabling extracellular stimuli to induce or modulate intracellular signaling in the brain endothelium [58,59,60]. We have previously shown that EVs derived from OSA-NC(+) induced BBB alterations in tight junction integrity zonula occludens-1 (ZO-1) protein cellular membrane topography compared to EVs derived from OSA-NC(−) children [27]. ZO-1 is one of several protein families that are critical for tight junction formation and maintenance, and it has now become apparent that stressful conditions can lead to dysfunction of brain endothelial tight junctions and consequently lead to adverse effects on cognition via EV-related biological activities affecting the BBB [61,62].

Plasma-derived EVs can induce the breakdown of the BBB [27,63]. However, considering the more expansive array of potential morbidities associated with OSA, it was unclear whether other endothelial cells originating from different vascular beds would exhibit similar susceptibility to circulating EVs or not. The current study illustrates for the first time the marked differences that exist among the various endothelial cell types in relation to the three phenotypes examined herein.

A major focus of endothelial biology is seeking to understand how endothelial cell barriers are formed and regulated in both health and disease [64]. Measurement of endothelial barrier resistance with ECIS allows for continuous impedance measurements to be monitored when cells are grown on monolayers. In this study, we took advantage of the unique functionalities offered by the ECIS technology to reveal differences in the susceptibility of various endothelial cell types to form a barrier, while being exposed to EVs derived from children with OSA and controls exhibiting different phenotypes of cognitive deficits. Here we show for the first time that plasma EVs in OSA children with neurocognitive deficits disrupt endothelial barrier function in naïve human primary BBB cells (hCMEC/D3) as well as in other endothelial cells, but that such susceptibility is cell-dependent (Figure 4). Our data show that circulating EVs in children with OSA-NC(+) adversely affect the BBB, and that such effect is clearly greater than EVs from OSA-NC(−), as illustrated by temporal impedance changes across the BBB cell monolayer. Such effects are also present in other vascular beds as tested in this study, suggesting that one of the major targets of circulating EVs in OSA, and particularly in OSA-NC(+), is the endothelium, a finding that is highly concordant with a multitude of previous studies in the field,. Indeed, we have previously shown that when cognitive deficits occur in children with OSA, they conspicuously overlap with perturbations in endothelial function as tested by laser Doppler flowmetry [52]. Furthermore, we now posit that identification of unique EVs cargo components may offer opportunities for future therapies and also serve as disease phenotype biomarkers, thereby permitting a more personalized and precise approach to patients with OSA [65]. EVs contain multiple components that are potentially biologically active (miRNA, proteins, and lipids) [66,67,68]. In subsequent studies, we will identify EVs contents that underlie the preponderant biological differences identified in our experiments. Although these studies did not identify which components of the EVs cargo underlie the functionally deleterious effects on the BBB, we hypothesize that differentially expressed cargo components, such as miRNAs [38,39], may play a pathophysiologically causal role in the induction of such neurocognitive deficits by disrupting the BBB.

Similarly, the wound-healing assay, which examines the recovery dynamics of endothelial cells, uses a standardized electrical approach to generate the wound in the confluent cell monolayers, rather than employs the more variable mechanical approaches [69]. The surrounding cells of the damaged circumscribed area then repopulate by migrating progressively toward the electrode and concurrently induce increases in the measured impedance over time, thus representing the actual rate of cell proliferation and migration. Addition of EVs derived from children with OSA-NC(+) reduced cell migration in some of the endothelial cell types compared to healthy children (CO) or children with OSA without cognitive deficits, indicating the extent of functional alterations associated with OSA in specific endothelial beds. Traditional wound-healing assays involve first growing a confluent cell monolayer, in which a small region is then purposefully damaged by the scratching of a line through the cellular layer with, for example, a needle. The image is then visually examined microscopically over time as the cells migrate and occupy the damaged area. This “healing” process will occur in a time frame of several hours to over a day contingent on the cell type, cell culture conditions, and the extent of the “wounded” region. However, the assay we adopted, which includes ECIS, provides automated and continuous information regarding changes in cell–substrate interactions and in cell morphology during wound-healing migration. Following the highly reproducible wounding process using a standardized electrical pulse, living cells located outside the sensing electrode will migrate and cover the wounded area until the wound is sealed, and such changes can be continuously reported by the ECIS system with a high degree of fidelity [70].

Elevated levels of hypoxia inducible proteins (HIF) are detectable in patients with OSA, and such proteins may also play a role in endothelial barrier disruption [71,72]. Future studies evaluating the presence and function of such proteins in EVs samples should assist in clarifying the specificity of EVs effects on endothelial cells. Such assessments were precluded in the present study due to the limited amount of plasma that was approved by the Ethics Committee in these children. Furthermore, in a similar study in adult subjects, we found that HIF-1 was activated in endothelial cells treated with EVs derived from untreated OSA patients compared to when such patients had received continuous positive airway pressure treatment for three months [44].

We should highlight some limitations in this study. First, the relatively small size of the sample of children studied will undoubtedly require additional studies involving more children, and potentially use pre- and post-treatment design rather than the cross-sectional approach we undertook herein. However, our results should promote the design and execution of larger studies by providing, based on current findings a priori, accurate estimates of statistical power. We should also highlight that despite the fact that the sample size per group was small, the subjects were selected from a large cohort (consisting of >600 children) and were carefully matched for gender, ethnicity, OSA severity, and BMI z-score. Second, the restricted quantities of plasma that were possible to obtain from the participants were dictated by the bioethical committee, and did not allow us to explore additional lines of investigation that would be pertinent as far as the unique differences reported heretofore.

## 4. Conclusions

Circulating plasma EVs in children with OSA, and more particularly among those with cognitive deficits, disrupt the integrity of the BBB by imposing adverse effects on endothelial barrier monolayer integrity and wound healing, while also exhibiting endothelial cell type selectivity. These could be due to differences in the conductivity of the monolayer via extracellular contacts in different types of endothelial cells (ECs). Circulating exosomal cargo may play important roles in the emergence of end-organ morbidity in pediatric OSA, and enhanced understanding of the significant pathological roles played by EVs and their cargo may offer opportunities for the development of novel therapeutic approaches.

## 5. Materials and Methods

### 5.1. Subject Characteristics

The study was approved by the human subject bioethics committee at The University of Chicago (protocol #09-115-B, July 2010–2018), and informed consent was secured from the legal caretaker of each participant. All approaches were in accordance with any of the relevant guidelines and regulations. To confirm eligibility of the participants, parents filled out a sleep questionnaire [73,74]. In this study, non-snoring children and snoring children were randomly selected and invited to participate (completed questionnaires). Our exclusion criteria were chronic medical conditions, genetic or craniofacial syndromes, developmental delays, a current Individual Education Plan at school indicative of significant learning or other difficulties, current use of psychotropic medications, and the presence of an acute infection. Children who met inclusion criteria were invited to the sleep laboratory for overnight polysomnography followed by extensive neurocognitive testing the next morning. Children were evaluated by trained psychometricians in a quiet room without a parent present. Psychometricians were unaware of the child’s sleep study and questionnaire findings. To ensure accuracy, double scores by the psychometricians were evaluated. Consecutive children with a diagnosis of OSA according to polysomnographic criteria and aged between 5 and 10 years were included. Assent was also obtained from children if they were >6 years of age. Control children were non-snoring children who had AHI < 1 h^−1^ TST in the overnight sleep study.

### 5.2. Overnight Polysomnography

All children underwent standard nocturnal polysomnography (NPSG) testing that included eight standard electroencephalogram (EEG) channels, bilateral electrooculograms, anterior tibial electromyography, two-lead electrocardiographic recordings, and oral and nasal airflow assessments with a thermistor, as well as a nasal pressure transducer, capnography, respiratory inductance plethysmography for chest and abdominal excursions, and oxyhemoglobin saturation by pulse oximetry that also included beat-by-beat pulse waveform. All signals were acquired with a commercially available system (Polysmith; Nihon Kohden America, Inc., Irvine, CA, USA), as previously described [75]. Sleep data were scored manually by pediatric PSG technicians according to the manual published by American Academy of Sleep Medicine (AASM) [75]. Oxygen desaturation index was defined as all events consisting of drops in SpO2 ≥ 3% divided by total sleep duration. The definition of arousal was strictly in accordance with the AASM Guidelines. The presence of OSA required an obstructive apnea–hypopnea index (AHI) ≥ 1 per hour of TST, and was based on the 2012 American Academy of Pediatrics consensus guideline [76].

### 5.3. Neurocognitive Assessments

In the morning immediately after NPSG, children were tested with the following test batteries: (a) Differential Ability Scales (DAS); (b) the Peabody Picture Vocabulary Test, Third Edition (PPVT-III); (c) the Expressive Vocabulary Test (EVT); and (d) the NEPSY (Developmental Neuropsychological Assessment) [12]. These tests have been extensively standardized and evaluate broad cognitive function and also specific domains of neuropsychological status. The tests are frequently used in educational and clinical settings for diagnostic purposes [12]. As previously described, cognitive deficits (NC(+)) were considered to be present if two or more cluster subtests were more than 1 SD below the mean [13]. All children underwent a blood draw the morning after the NPSG under fasting conditions.

### 5.4. Plasma and EVs Isolation

Blood samples were collected from 8:00 to 9:00 a.m. in Vacutainers tubes, and samples were centrifuged for 2000× *g* for 20 min at 4 °C. The supernatant was collected and stored at −80 °C for further analysis. EVs were isolated from plasma using the Total EVs Isolation Kit according to the manufacturer’s protocol (Life Technologies, Carlsbad, CA, USA). Briefly, plasma was centrifuged at 2000× *g* for 20 min to remove cell/debris. The supernatants were collected and 0.2 volume of the Total EVs Isolation Reagent (TEIR) was added. The mixtures were incubated with TEIR at 4 °C for 30 min, further filtered and through 0.22 µm, and followed by centrifugation at 10,000× *g* for 10 min, and pellets were solubilized in 1× phosphate-buffered saline (PBS) [38].

### 5.5. EVs Characterization and Quantification

Plasma EVs size distribution was evaluated using electron microscopy (model Tecnai F30 at 300 KV; FEI company Hillsoro, Oregon, USA). EVs were placed on formvar-carbon-coated electron microscopy grids, and allowed to stand for 5–10 min for EVs adsorption. Grids with adherent EVs were transferred to three 25 μL drops of DPBS for washing, fixed with 2% paraformaldehyde for 7 min, then incubated with 25 μL drops of 2% uranyl acetate and examined by electron microscopy. Size distribution of EVs was assessed and quantified as previously described [38].

EVs were quantified using enzymatic fluorescent assay (FluoroCet #FCET96A quantitation kit; System Biosciences, Mountain View, CA, USA) according to the manufacturer’s protocols [32]. The assay was used to measure the esterase activity of cholesteryl ester transfer protein (CETP) activity, and the standard curve was calibrated to known EVs counts. Enzyme activity of the samples and the standards were determined by incubation at room temperature for 20 min. Fluorescence was measured with a fluorescence plate reader (GloMax 9301 Multi-detection System; Promega, Madison, WI, USA) immediately at excitation (530–570 nm) and emission (590–600 nm). This kit does not differentiate between exosomes or EVs; therefore, we cannot eliminate the possibility that there are other extracellular particles in the samples, albeit in small quantity.

### 5.6. Cell Cultures

Primary cultures of human endothelial cells derived from multiple vascular beds were used in this study. Human cerebral microvascular endothelial cells (HCMEC/D3) were purchased from Sigma-Aldrich (Sigma-Aldrich, Millipore, St. Louis, MO, USA), and cells were maintained following the manufacturer’s instructions. The cells were grown in cultured media of EndoGRO™-MV Complete Media Kit (Cat. No. SCME004, Sigma-Aldrich) supplemented with 1 ng/mL FGF-2 (Cat. No. GF003, Sigma-Aldrich). Human coronary artery endothelial cells (HCAEC), human iliac artery endothelial cells (HIAEC, Lonza, Walkersville, MD, USA), human dermal microvascular endothelial cells (hMVEC-d), human cardiac microvascular endothelial cells (HMVEC-C), and human lung microvascular endothelial cells (HMVEC-L) were purchased from Lonza (Walkersville, MD, USA), and cells were maintained following the manufacturer’s instructions. For cell culture, the procedures consisted in use of endothelial growth medium (EGM™-2MV BulletKit™; Clonetics) supplemented with 5% fetal bovine serum (FBS; Clonetics) and cells were incubated at 37 °C and 5% CO_2_. For serial passaging (always before passage 4), the cells were dissociated by trypsinization, centrifuged at 220× *g* for 7 min, and replated at the correct density.

### 5.7. Plasma and Exosomes Isolation

Blood samples were collected from 8:00 to 9:00 a.m. in Vacutainers tubes, and samples were centrifuged for 2000× *g* for 20 min at 4 °C. The supernatant was collected and stored at −80 °C for further analysis. Exosomes were isolated from plasma using the Total Exosome Isolation Kit according to the manufacturer’s protocol (Life Technologies, Carlsbad, CA, USA). Briefly, plasma was centrifuged at 2000× *g* for 20 min to remove cell/debris. The supernatants were collected and 0.2 volume of the Total Exosome Isolation Reagent (TEIR) was added. The mixtures were incubated with TEIR at 4 °C for 30 min, further filtered and through 0.22 µm, and followed by centrifugation at 10,000× *g* for 10 min, and pellets were solubilized in 1× phosphate-buffered saline (PBS) [38].

### 5.8. EVs Endothelial Cell Uptake

EVs were labeled using PKH67-GL (Sigma-Aldrich), and images were acquired using confocal microscopy. EVs were labeled for 10 min at 37 °C and the mixtures were precipitated with ExoQuick-TC reagents (System Biosciences), then incubated on ice for 30 min, centrifuged at 4 °C at 13,000 rpm, and filtered to remove unbound dyes. The pellets were suspended in 1× PBS buffer, and the labeled EVs were placed on confluent coverslips of hCMEC/D3 (Sigma-Aldrich, Millipore, CA, USA) for 24 h in a cell culture incubator at 37 °C. The labeled PKH67-Green color was imaged with a Leica SP5 Tandem Scanner Spectral 2-photon confocal microscope (Leica Microsystems, Inc., Buffalo Grove, IL, USA) with a 63× oil-immersion lens. As the negative control, PKH67-Green was prepared with all reagents, but did not include EVs, and thus ascertained for any unincorporated dyes that may be carried over after centrifugation. Nuclei were imaged by staining with Hoechst 33,342 (Sigma-Aldrich) at a concentration of 1 µg/mL in PBS for 5 min.

### 5.9. Flow Cytometry

To analyze for selective subpopulations of EVs surface markers, Evs were incubated with Exo-Flow™ kits (System Biosciences, Mountain View, CA, USA) and then subjected to FACS analysis (FACSCalibur, BD Biosciences, San Jose, CA, USA). Briefly, Evs were incubated with commercially available magnetic beads of 9.1 nm diameter that incorporated different Evs markers including tetraspanins (#EXOFLOW150A-1, CD9, CD63, and CD81). EVs and the magnetic beads were incubated for 12 h at 4 °C according to the manufacturer’s manual. Two negative controls were also carried out, with negative #1 (all reagents without antibodies and no EVs) and negative #2 (all the reagents and beads but without EVs). In the FACS, 30,000 events were acquired and then analyzed using FlowJo Software (Tree Star, Inc., Ashland, OR, USA). The average of MFI for negative #1 was used to normalize the samples with and without EVs.

### 5.10. Electric Cell–Substrate Impedance Sensing (ECIS)

Real-time change in transendothelial monolayer electrical resistance was measured using the ECIS system (http://www.biophysics.com/products-ecisz0.php). Endothelial cells originating from the multiple vascular beds were grown as described above in media containing depleted FBS for 24 h. The ECIS assays were conducted using the 8-well ECIS arrays (PC; 8W10E) via the ECIS-Z station. The arrays (8W10E) were treated with 10 mM l-cysteine (Sigma-Aldrich, Millipore, St. Louis, MO, USA) followed by coating with Collagen Type II (Sigma-Aldrich, Millipore, St. Louis, MO, USA). A total of 50,000 cells were plated and grown to confluence into ECIS arrays as a single confluent monolayer. ECIS assessments were performed using the multiple frequency/time (MFT) option to record continuously changes in impedance over a broad spectrum of frequencies. When impedance signals stabilized and therefore indicated that a confluent monolayer and a functional barrier had formed, EVs were added in duplicated wells and placed into the ECIS instrument for continuous monitoring up to 24 h. As cultured cells adhere and spread on the electrode surface, impedance changes, and such changes over time serve as a measure of the disruption of the endothelial cellular junction. Control reference values were established by using culture medium (500 μL/well) alone, and then compared with the values recorded when electrodes were covered with a monolayer of cells in 500 μL medium.

### 5.11. Wound-Healing Assay

Electric cell–substrate impedance sensing (ECIS) was used as above to monitor the recovery of endothelial cells in a real-time fashion after wound healing [77]. Wounds to the endothelial cell monolayers were created by applying a burst of high-intensity electrical current while cells were maintained in a humidified 5% CO_2_ incubator at 37 °C. ECIS measurements included three steps: (a) creating confluent cell layers, (b) wounding the cells that were aligned on the small gold electrode, and (c) examining the healing process by evaluating impedance. Rather than disrupting the cell layer using the traditional “scrape”, the ECIS wounding approach employs electric current (i.e., the “electrical shock”) to wound the cells and monitor the subsequent healing process. The arrays (8W10E) were treated with l-cysteine and with Collagen Type II as described above. ECIS was performed while recording the multiple frequency/time (MFT) option to evaluate impedance changes over a broad spectrum of frequencies. Before starting ECIS measurements, 300 μL medium containing 5% depleted bovine albumin serum (BAS) were placed in each well and were allowed to stabilize for ≈30 min, after which 200 μL of cell suspension (5 × 10^5^ cells per mL) were added to each well. After cell inoculation, the wells were incubated for 24 h, and once they reached confluence, wounding was carried out with the integrated electrical field module (3500 μA, 20 s at 48 kHz) for a total time of 5 min. EVs were applied to the designated wells. The healing process was monitored continuously as cells migrated and proliferated onto the electrode. For a quantitative comparison of the different cell lines, we monitored the duration required for capacitance to recover. The ECIS wounding assay is an automated assay, and both cell wounding and measurements of the following healing process were performed under computer control.

### 5.12. Data Analysis

All results are presented as means ± standard deviation (SD). Analyses were conducted using SPSS software (version 21.0; SPPS Inc., Chicago, IL, USA). Comparisons between EVs-treated and untreated cells were performed by evaluating the effect of treatment on an endothelial monolayer using repeated measures two-way ANOVA with multiple comparisons (main mean effect), followed by post hoc Tukey tests. A level of *p* ≤ 0.05 was considered statistically significant for all analyses.

## Figures and Tables

**Table 1:** Demographic and polysomnographic findings among OSA children with either present (*n* = 12) or absent (*n* = 12) cognitive deficits and control children (CO; *n* = 6).

Variable	CO (*n* = 6)	OSA-NC(−) (*n* = 12)	OSA-NC(+) (*n* = 12)
Age (years)	6.3 ± 1.7	6.3 ± 1.6	6.5 ± 1.4
Gender (male, %)	50.0	50.0	50.0
Ethnicity (African American, %)	66.7	66.7	66.7
BMI z-score	1.02 ± 0.29	1.06 ± 0.25	1.04 ± 0.26
Total sleep duration (min)	473.2 ± 75.1	474.2 ± 66.3	478.8 ± 67.1
REM sleep (%)	20.2 ± 10.2	17.9 ± 9.5	18.2 ± 8.1
Sleep latency (min)	24.6 ± 15.2	20.7 ± 13.9	22.4 ± 15.6
REM latency (min)	118.7 ± 67.4	119.5 ± 52.4	117.7 ± 49.5
Total Arousal Index (events/hour TST)	12.5 ± 8.6 *	20.9 ± 10.3	22.8 ± 11.4
Respiratory Arousal Index (events/hour TST)	0.4 ± 0.2 *	8.2 ± 4.7	7.9 ± 4.2
Obstructive Apnea Hypopnea Index (events/hour TST)	0.5 ± 0.3 *	19.4 ± 6.9	19.9 ± 7.1
SpO_2_ Nadir (%)	94.1 ± 1.3 *	82.1 ± 9.0	80.3 ± 8.6
ODI3%	0.4 ± 0.2 *	19.0 ± 8.4	18.4 ± 7.7

ODI3% - oxyhemoglobin desaturation index 3%; Data are expressed as mean ± SD. * *p* < 0.001 (OSA vs. CO).

**Table 2:** Effects of plasma exosomes derived from healthy (CO), OSA-NC(−), and OSA-NC(+) children on wound healing recovery (%) using endothelial cells originating from multiple diverse vascular beds.

Endothelial Cell Line	No Exosomes	CO	OSA-NC(−)	OSA-NC(+)
hCMEC/D3	100	97.6 ± 5.23	71.87 ± 5.37	45.08 ± 3.59 **
hMVEC-d	100	98.95 ± 6.63	82.97 ± 5.64	79.92 ± 4.25*
hCAEC	100	93.07 ± 6.89	86.50 ± 6.78	83.82 ± 6.71
hIAEC	100	96.98 ± 7.21	95.15 ± 7.35	98.29 ± 7.11
hMVEC-C	100	97.02 ± 8.01	91.63 ± 6.86	86.83 ± 5.24
hMVEC-L	100	88.62 ± 6.84	86.07 ± 6.29	87.87 ± 6.29

All data are expressed as mean ± SD. OSA-NC(+) vs. CO or OSA-NC(−); * *p* < 0.05; ** *p* < 0.01.

## Abbreviations

OSA	Obstructive sleep apnea
BBB	Blood–brain barrier
PSG	Polysomnography
OSA-NC(−)	OSA without cognitive deficits
OSA-NC(+)	OSA with cognitive deficits
AHI	Apnea–hypopnea index
HCAEC	Human coronary artery endothelial cells
HIAEC	Human iliac artery endothelial cells
HMVEC-D	Human dermal microvascular endothelial cells
HMVEC-C	Human cardiac microvascular endothelial cells
HMVEC-L	Human lung microvascular endothelial cells
hCMEC/D3	Human brain microvascular endothelial cell
ECIS	Electric cell–substrate impedance sensing
BMI	Body mass index
CNS	Central nervous system
FMI	Median, fluorescence intensity
